# Low rates of headache and migraine associated with intravenous immunoglobulin infusion using a 15-minute rate escalation protocol in 123 patients with primary immunodeficiency

**DOI:** 10.3389/fimmu.2022.1075527

**Published:** 2023-02-02

**Authors:** Bob Geng, Kim Clark, Mark Evangelista, Eric Wolford

**Affiliations:** ^1^ Division of Allergy & Immunology, University of California, San Diego, CA, United States; ^2^ Global Medical Department, Bio Products Laboratory, Ltd., Elstree, United Kingdom; ^3^ Biostatistics Department, Atlantic Research Group, Charlottesville, VA, United States

**Keywords:** IVIg, headache, migraine, primary immunodeficiency, rate escalation, pooled

## Abstract

**Introduction:**

Headache and migraine adverse events are common concerns in the administration of intravenous immune globulins (IVIG). Trials of IVIG for primary immunodeficiency (PI) are typically small and have reported headache and migraine data inconsistently.

**Methods:**

We analyzed headache and migraine in pooled data from three pivotal trials of Gammaplex^®^ 5% and 10% in PI (NCT00278954 from January 18, 2006; NCT01289847 from January 27, 2011; NCT01963143 from September 13, 2013). The trials were pooled in a retrospective analysis that included two 12-month open-label non-comparative trials of the 5% IVIG product and one 6-month open-label crossover bioequivalence trial comparing the 5% IVIG and 10% IVIG products. The population included adult and pediatric patients, who received IVIG infusions of 300-800 mg/kg/infusion every 21 or 28 days using a 15-minute rate escalation protocol.

**Results:**

In total, 1482 infusions were administered to 123 patients, with 94.6% of infusions achieving the maximum infusion rate. At least one product-related headache was reported in 6.1% (90/1482) of infusions. At least one product-related migraine was reported in 0.5% (7/1482) of infusions. Headache rates were higher for adults vs pediatric patients, females vs males, and 21-day vs 28-day dosing schedules, but were similar for the 5% and 10% IVIG products. Most headaches and migraines occurred during or within 72 hours of the infusion. Rates decreased after the first few infusions.

**Discussion:**

Patients receiving this IVIG product on a 15-minute rate escalation protocol had low rates of headache and migraine for both the 5% and 10% formulations.

## 1 Introduction

Intravenous immune globulin (IVIG) is a standard therapy for the treatment of primary immunodeficiency disorders (1–3). IVIG may be administered across a broad range of infusion rates depending on the formulation, product labeling, and patient tolerance (4–6). Minimizing the time required for IVIG administration may benefit patient quality of life and reduce healthcare utilization. However, infusion rates may be limited by rate-related adverse events (AEs) (2, 7–10). Headache is among the most common AEs reported with IVIG infusion, although reported rates of headache and other AEs vary considerably. Reported migraine events are more severe than other headaches but much less common and often delayed (6, 11–15).

Gammaplex^®^ (immune globulin intravenous [human]) is an intravenous immune globulin available in both 5% and 10% formulations. In the United States, both formulations are indicated for treatment of patients with primary immunodeficiencies (PI) and chronic immune thrombocytopenic purpura (ITP), and have been investigated in four pivotal clinical trials, three in PI and one in ITP (16–21). In all four studies, including the high-dose ITP trial, a 15-minute rate escalation infusion protocol was used with an overall positive tolerability profile with low rates of headache and migraine (18–20).

Because PI is rare, trials of IVIG in patients with PI tend to be small, with most late-phase clinical trials of these agents involving 50 or fewer patients (18, 20, 22–33). While these trials produce sufficient data to satisfy FDA requirements for safety and efficacy, the overall data sets are limited. One way to evaluate a larger data set, to either reaffirm what is already known or to investigate new questions, is to conduct an analysis using pooled data from multiple studies (34). In this report, we evaluated pooled data from the three registrational clinical trials of Gammaplex in PI to determine overall rates of headache and migraine while following a 15-minute infusion protocol. There have been few such pooled analyses for IVIG or subcutaneous immunoglobulin (SCIG) products (35–37) and this is the first such analysis of Gammaplex.

## 2 Materials and methods

This was an exploratory retrospective analysis of data pooled from three pivotal trials of the IVIG products Gammaplex 5% and Gammaplex 10%, (immune globulin intravenous [human], Bio Products Laboratory, Elstree, UK) for the treatment of PI. **Table 1** summarizes key characteristics of the trials. All three trials were open-label studies during which patients received IVIG at a total dose of 300-800 mg/kg/infusion. Patients received infusions every 21 or 28 days on the same schedule as their pre-trial regimen. The patient populations and treatments varied across studies. In studies GMX01 and GMX04, adult and pediatric patients respectively received 5% IVIG for one year. In study GMX07, adults received at least five doses of 5% IVIG and at least five doses of 10% IVIG in a crossover design, while pediatric patients received five doses of 10% IVIG (18–20). Adverse events including headache and migraine were identified by patient self-report in patient interviews and/or a patient diary, and were coded using Medical Dictionary for Regulatory Activities (MedDRA) v8.1 (studies GMX01 and GMX04) and v17.0 (GMX07) (18–20).

**Table 1:** Characteristics of the pooled trials (18–20).

Study	Moy 2010 (GMX01)	Melamed 2016 (GMX04)	Wasserman 2017 (GMX07)
Design	Open-label non-comparative	Open-label non-comparative	Open-label crossover bioequivalence
Patient Population	Adult and pediatric ≥3 years	Pediatric 2-16 years	Adult and pediatric 2-55 years
Treatment	IVIG (5% formulation)	IVIG (5% formulation)	IVIG (5% or 10% formulations)
Duration of Treatment	12 months	12 months	6 months (Adults: ≥5 infusions of each formulation; pediatric patients: ≥5 infusions of the 10% formulation)
Dose	300-800 mg/kg/infusion every 21 or 28 days		

This retrospective pooled analysis did not directly engage any patients. All trials included in the analysis were approved by an institutional review board/ethics committee at each study center and obtained written informed consent from each participant (or their parent/guardian, if applicable) to participate in the study. Participants from the included studies agreed to have their results published.

### 2.1 Statistical analysis

Infusion and adverse event data were pooled across three Gammaplex studies (GMX01, GMX04, GMX07). Summaries of infusion protocol were generated to identify the proportion of infusions following the 15-minute rate escalation protocol and reaching the maximum infusion rate. Adverse events were documented based on direct observation during each infusion, interviews with the patient, and/or diary entries. Summaries of tolerability were generated to identify the proportion of infusions associated with product-related AEs. Product-related AEs were defined as those considered possibly, probably, or definitely related to administration of the product by the investigator. Infusion-associated AEs were defined as those occurring during or within 72 hours of the end of the infusion, specifically headaches and migraines. When assessing infusion protocol and tolerability, 95% confidence intervals for the proportion of infusions were calculated using the Clopper-Pearson (exact) method. Differences in tolerability across product formulation, dose categories, infusion order, and PI diagnosis were summarized using a 95% confidence interval for difference of proportions. All analyses were produced using SAS 9.4. All *p*-values are descriptive.

Exploratory subgroup analyses were conducted by gender (male vs female), age (adult vs pediatric), product formulation (5% IVIG vs 10% IVIG), infusion schedule (every 21 days vs every 28 days), and dose tertile (≤429 mg/kg, >429-≤526 mg/kg, and >526 mg/kg). Exploratory bivariate analyses were conducted for age and product formulation, age and gender, and gender and product formulation. Data were analyzed both by infusions and by patients to evaluate the extent to which a subset of patients accounted for headache and migraine events.

### 2.2 Infusion rate

In all trials, infusion of the IVIG product was started at an initial infusion rate for 15 minutes (0.01 mL/kg/min for the IVIG 5% formulation, 0.005 mL/kg/min for the IVIG 10% formulation), then advanced every 15 minutes if tolerated to a maximum of 0.08 mL/kg/min, using the protocols shown in **Table 2**. The infusion rate could be reduced or interrupted based on the investigator’s clinical judgment if any AE of moderate to severe intensity occurred and resumed at the investigator’s discretion at a rate tolerated by the patient. The difference in initial infusion rate by formulation was to ensure an equivalent protein content was administered during the comparative registration trial.

**Table 2:** Infusion rate protocols (18).

	IVIG 5% Formulation			IVIG 10% Formulation		
	mL/kg/ min	mg/kg/ hour	Elapsed Time (min)	mL/kg/ min	mg/kg/ hour	Elapsed Time (min)
Start	0.01	30	0-15	0.005	30	0-15
Increments	0.02	60	16-30	0.01	60	16-30
	0.04	120	31-45	0.02	120	31-45
	0.06	180	46-60	0.04	240	46-60
	0.08	240	61 to end	0.06	360	61-75
				0.08	480	76 to end

## 3 Results

The analysis included all Gammaplex infusions administered during the PI clinical trials (1482 infusions administered to 123 patients). Of these, 1234 infusions of the 5% IVIG formulation were administered to 108 patients and 248 infusions of the 10% IVIG formulation were administered to 47 patients. Both adult and pediatric patients participated in the 5% and 10% studies. Key patient, disease, and treatment characteristics for each trial are summarized in **Table 3**.

**Table 3:** Patient Demographics, Disease Characteristics, and Infusion Parameters (18–20).

Characteristic	Moy 2010 (GMX01)	Melamed 2016 (GMX04)	Wasserman 2017 (GMX07)	Pooled Data
Patients, n	50	25	48	123
Male, n (%)	26 (52.0)	19 (76.0)	20 (41.7)	65 (52.8)
Age, years				
Mean ± SD	44.0 ± 19.1	10.4 ± 3.8	30.1 ± 17.3	31.7 ± 20.5
Median (Range)	44.5 (9, 78)	11.0 (3, 16)	30.5 (3, 55)	30.0 (3, 78)
Age range, n (%)				
2-5	0	3 (12.0)	2 (4.2)	5 (4.1)
6-11	2 (4.0)	12 (48.0)	7 (14.6)	21 (17.1)
12-15	2 (4.0)	7 (28.0)	6 (12.5)	15 (12.2)
16-34	11 (22.0)	3 (12.0)	12 (25.0)	26 (21.1)
35-49	15 (30.0)	0	12 (25.0)	27 (22.0)
≥50	20 (40.0)	0	9 (18.8)	29 (23.6)
Ethnicity, n (%)				
Caucasian	46 (92.0)	25 (100)	45 (93.8)	115 (93.5)
African-American	2 (4.0)	0	0	2 (1.6)
Hispanic	2 (4.0)	0	3 (6.3)	5 (4.1)
Diagnosis, n (%)				
CVID	46 (92.0)	22 (88.0)	38(79.2)	105 (85.4)
XLA	4 (8.0)	3 (12.0)	8 (16.7)	14 (11.4)
Other PI	0	0	2 (4.2)	3 (2.4)
Treatment Parameters				
Infusions, n	703	368	411	1482
Premedicated, n (%)	0	0	35 (8.5)*	35 (2.4)*
IVIG Formulation, number of infusions				
5%	703 (100%)	368 (100%)	163 (39.7%)	1234 (83.3%)
10%	0	0	248 (60.3%)	248 (16.7%)

CVID, common variable immunodeficiency; PI, primary immunodeficiency; XLA, X-linked agammaglobulinemia.

*Three adults were premedicated for a total of five infusions of the 5% IVIG formulation and for five infusions of the 10% IVIG formulation. Six pediatric patients were premedicated for a total of 25 infusions of the 10% IVIG formulation. Given the small number of patients and infusions, the effects of premedication were not evaluated in this study.

### 3.1 Achievement of the maximum infusion rate

**Figure 1:**
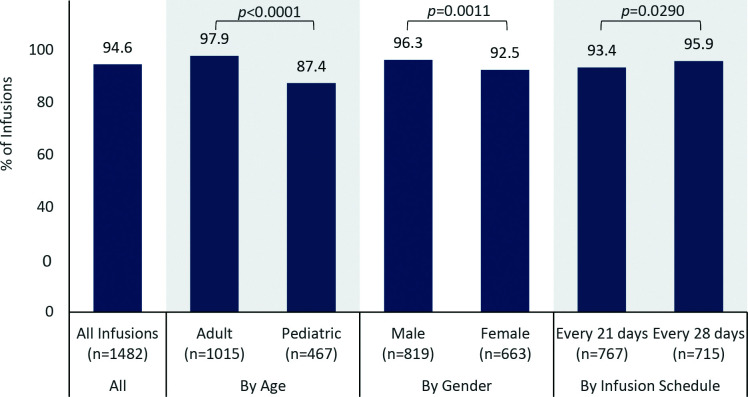
Percentage of infusions in which the maximum infusion rate was achieved, by subgroup.

The maximum infusion rate was achieved in 94.6% (1402/1482) of infusions (**Figure 1**). The probability of reaching the maximum infusion rate was higher for adults than pediatric patients (97.9% vs 87.4%, *p *< 0.0001), higher for males than for females (96.3% vs 92.5%, *p *=* *0.0011), higher for the 5% IVIG formulation than for the 10% IVIG formulation (96.6% vs 84.7%, *p *<* *0.0001), and lower for patients receiving infusions every 21 days than for patients receiving infusions every 28 days (93.4% vs 95.9%, *p *=* *0.0290). Although statistically significant, these differences are likely explained by large sample sizes within groups and are unlikely to represent clinically meaningful differences. Breaking down the results by age and product formulation, the probability of achieving the maximum rate was not different for the 5% and 10% IVIG formulations in adults (98.2% vs 96.4%, *p *=* *0.1354), but was higher for the 5% formulation than the 10% formulation in pediatric patients (93.0% vs 61.0%, *p *<* *0.0001).

### 3.2 Percentage of infusions with reported product-related headache or migraine

**Figure 2:**
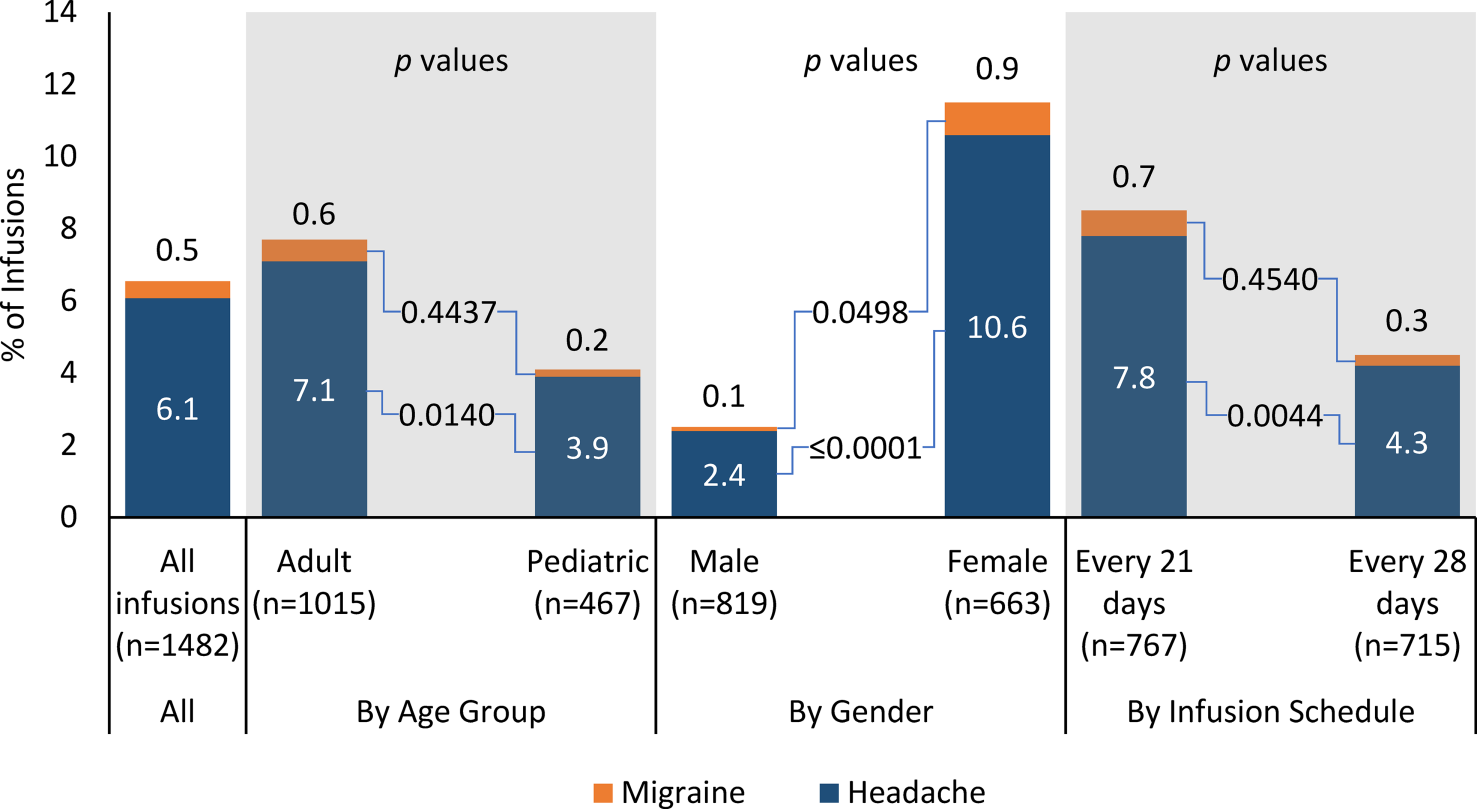
Percentage of infusions associated with product-related headache or migraine by subgroup.

Product-related, infusion-associated headache was reported in 6.1% (90/1482) of all infusions (**Figure 2**). The percentage of infusions with at least one product-related headache was higher for adults than for pediatric patients (7.1% vs 3.9%, *p *=* *0.0140), lower for males than females (2.4% vs 10.6%, *p *<* *0.0001), similar for the 5% IVIG product and the 10% IVIG product (6.0% vs 6.5%, *p *=* *0.7711), and higher for patients receiving infusions every 21 days vs patients receiving infusions every 28 days (7.8% vs 4.2, *p *=* *0.0044).

In the age-by-product breakdown, the percentage of infusions with at least one product-related, infusion-associated headache was similar for the 5% IVIG product and the 10% IVIG product in adults (7.1% vs 7.2%, *p *=* *0.8702) and for pediatric patients (3.6% vs 4.9%, *p *=* *0.5359). In the age-by-gender breakdown, infusions with headache were less likely with adult males than adult females (2.0% vs 11.8%, *p *<* *0.0001), but rates were similar for pediatric males and pediatric females (3.0% vs 5.8%, *p *=* *0.1901).

Product-related, infusion-associated migraine was reported in 0.5% (7/1482) of all infusions (**Figure 2**). In subgroup analyses, the percentage of infusions with at least one product-related, infusion-associated migraine was similar for adults and pediatric patients (0.6% vs 0.2%, *p *=* *0.4437), lower for males than females (0.1% vs 0.9%, *p *=* *0.0498), similar for the 5% IVIG product and the 10% IVIG product (0.3% vs 1.2%, *p *=* *0.0963), and similar for patients receiving infusions every 21 days vs patients receiving infusions every 28 days (0.7% vs 0.3%, *p *=* *0.4540).

### 3.3 Percentage of patients reporting at least one product-related headache or migraine

Headache and migraine were reported by 28.5% (35/123) and 4.1% (5/123) patients, respectively. The percentage of patients reporting at least one product-related headache was similar for adults and pediatric patients (28.0% [23/82] vs 29.3% [12/41], *p *=* *0.9999). Most adult and pediatric patients did not report a headache (72.0% [59/82] and 70.7% [29/41], respectively). Adults reported headache in 72 infusions. Four infusions were associated with two headaches each, for a total of 76 headaches. Pediatric patients reported headaches in 18 infusions. One infusion was associated with two headaches, for a total of 19 headaches.

The percentage of patients reporting at least one headache was lower for males than females (18.5% [12/65] vs 39.7% [23/58], *p *=* *0.0156), showed a non-significant trend toward higher rates with the 5% IVIG formulation than the 10% IVIG formulation (29.6% [32/108] vs 14.9% [7/47], *p *=* *0.0693), and higher for patients receiving infusions every 21 days vs patients receiving infusions every 28 days (38.6% [22/57] vs 19.7% [13/66], *p *=* *0.0274). Breaking down the results by age and product formulation, the percentage of patients reporting at least one product-related headache was non-significantly larger for the 5% IVIG formulation than for the 10% IVIG formulation in adults (28.0% [23/82] vs 12.5% [4/32], *p *=* *0.0911), and for pediatric patients (34.6% [9/26] vs 20.0% [3/15], *p *=* *0.4799). Adult males were less likely to report at least one headache than adult females (10.8% [4/37] vs 42.2% [19/45], *p *=* *0.0026), but rates were similar for pediatric males vs pediatric females (28.6% [8/28] vs 30.8% [4/13], *p *=* *0.9999). The percentage of patients reporting at least one headache did not differ markedly across dose tertiles (28.3% [13/46], 28.6% [14/49], and 19.6% [9/46] for doses ≤429 mg/kg, >429-≤526 mg/kg, and >526 mg/kg respectively).

At least one product-related migraine was reported by 4.1% (5/123) of patients. Of the seven reported migraines, six were reported by four adult female patients, with four migraines associated with infusion of the 5% IVIG formulation and two with the 10% IVIG formulation. The remaining migraine was reported by one male pediatric patient who was receiving the 10% IVIG formulation. The percentage of patients reporting at least one product-related migraine was significantly lower for males than females (0% [0/37] vs 8.9% [4/45], *p *=* *0.1229, but was not significantly different for any other subgroup comparisons.

### 3.4 Timing of headache and migraine events

**Figure 3:**
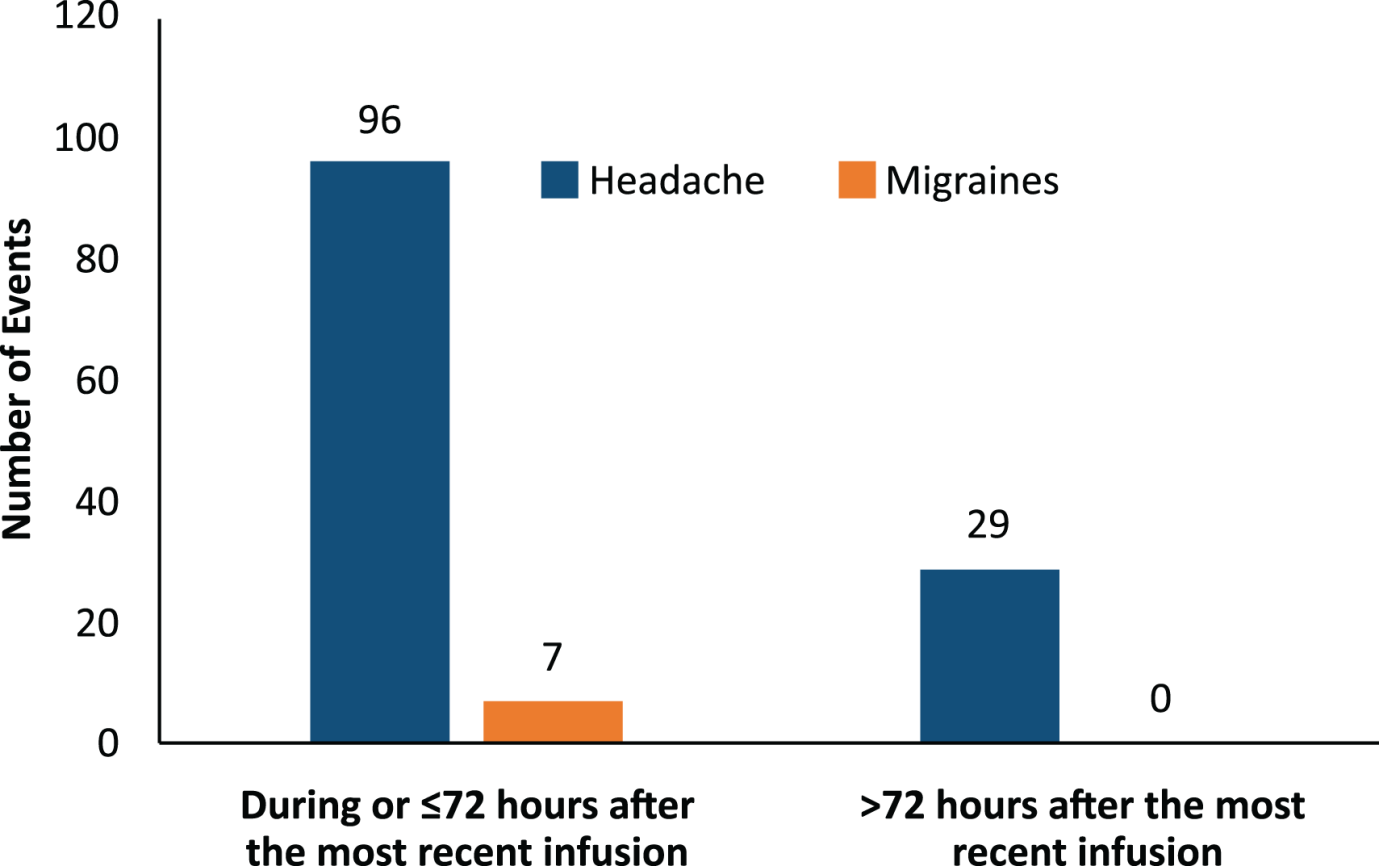
Timing of migraine and headache relative to the start of the infusion.

**Figure 4:**
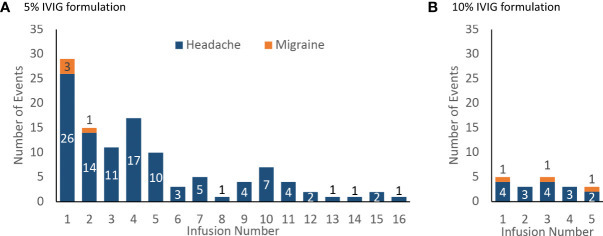
Incidence of product-related headache and migraine by infusion number, number of events **(A)** 5% IVIG formulation (108 patients for infusions 1–5, then 75 patients for infusions 6–18) **(B)** 10% IVIG formulation (48 patients).

Most headache events (77%, 96/125) and all migraine events (100%, 7/7) occurred within 72 hours of the start of the infusion (**Figure 3**). Most headaches and migraines occurred in the first few IVIG infusions for the 5% IVIG formulation (**Figure 4A**). No clear temporal pattern was apparent for the 10% formulation (**Figure 4B**).

## 4 Discussion

Headache is clearly associated with IVIG therapy for PI, but not with PI itself. In this retrospective analysis, we analyzed pooled data from three clinical trials of a single IVIG agent to confirm and extend our understanding of headache and migraine associated with IVIG infusions. Our analysis provides a systematic evaluation of infusion times and rates of headache and migraine in a large population of patients receiving IVIG treatment for PI. Pooled analyses can be used to reaffirm data present in individual trials or to understand new concepts that may be better represented in a larger data set (35–37). To our knowledge, this is the first such analysis of pooled data from multiple pivotal trials of an IVIG therapy.

Almost all patients achieved the maximum infusion rate specified in the product labeling, utilizing an infusion protocol with 15-minute rate escalation increments. Recent trials of other IVIG products have reported achieving per-protocol infusion rates in >90% of infusions, but with longer rate escalation increments (30 minutes vs 15 minutes for the IVIG formulations used in this analysis) (22, 24). In our study, subgroup analyses demonstrated differences in achievement of the maximum infusion rate by groups, with higher probabilities of achieving the maximum rate for adults vs pediatric patients, males vs females, and infusions every 28 days vs every 21 days. While these differences reached statistical significance likely due to a large number of infusions, they do not appear to represent clinically meaningful differences. The percentage of patients who achieved the maximum infusion rate was significantly different between the 5% and 10% formulations for pediatric patients but not for adult patients. Among pediatric patients, those receiving the 5% IVIG formulation were more likely to reach the maximum rate than those receiving the 10% IVIG formulation. This difference is not explained by reported AEs, including headaches or migraines. Of the 32 infusions in this group that did not reach maximum infusion rate, only six were associated with a product-related adverse event, including only two associated with product-related headache. None were associated with migraine. Other potential reasons for not achieving the maximum rate were not documented.

The observed rates of product-related headache (6.1% of infusions, 28.5% of patients) and migraine (0.5% of infusions, 4.1% of patients) occurred with 94.6% of patients achieving the maximum infusion rate, and use of a 15-minute rate escalation protocol. To place these results in context, headache rates reported in recent studies of IVIG in PI range from 2% to 22% on a per-infusion basis, and from 8% to 50.8% on a per-patient basis (13, 22, 24–26, 32, 38). Our results cannot be compared directly with these trials due to differences in trial design, patient population, and other factors. That said, our results suggest that the 15-minute rate escalation protocol is a clinically reasonable option with these products. Rates of product-related headache were significantly higher for adults than for pediatric patients at the infusion level but not on a per-patient basis, suggesting that adults were more likely to report multiple headache events than pediatric patients. Consistent with previously published studies, rates of headache were significantly higher for adult females than adult males at the infusion level and on a per-patient basis (6, 14). However, in the pediatric subgroup, headache rates were similar for males and females. Although the number of migraine events was small, adult females had significantly higher rates of migraine than adult males. The difference between males and females was apparent for both the 5% and 10% IVIG formulations.

Despite short (15-minute) escalation increments in the infusion protocol, rates of product-related headache were not significantly different for the 5% and 10% formulations in the entire population, the adult subgroup, and the pediatric subgroup, whether calculated at the infusion level or on a per-patient basis. In contrast to our results, 10% IVIG formulations have historically been associated with increased adverse event rates compared to 5% IVIG formulations (2, 6, 11). Patients receiving IVIG on a 21-day schedule had higher headache rates than those receiving it on a 28-day schedule. In the authors’ historical experience, there has been a tendency to limit the rate of IVIG administration and an assumption that less concentrated IVIG formulations are less likely to cause adverse reactions (eg, 5% less likely than 10% IVIG). There is little comparative data to support this assumption, and our results suggest that shorter rate escalation protocols and use of 10% formulations are feasible for many patients.

With IVIG therapy, first doses have been associated with more reported AEs than subsequent doses (2, 14, 26). In our study, most headaches and migraines occurred within 72 hours of the infusion and most occurred in association with the first few infusions, with rates decreasing over time. Although adverse reactions to IVIG can occur at any point, these findings support the standard practice of heightened concern for AEs in the first few infusions. This decrease in headache rates after the first few doses implies that per-infusion headache rates (driven by the long-term average) will be considerably lower than per-patient headache rates (driven by the first few doses) in studies that report on multiple IVIG infusions.

Product-related headache and migraine were concentrated in a minority of patients, and only a small proportion of the total number of infusions were preceded with premedications. This finding should serve as a reminder that many patients may tolerate these infusions without difficulty and without the need for premedications. Perhaps greater emphasis on selecting patients most at risk for AEs would result in more judicious use of premedication.

Rapid IVIG infusion is generally considered to be a risk factor for IVIG-related headache (13). One retrospective study found an association between slow infusion and increased headache; however, this association may have occurred because the infusion rate was slowed when patients developed headache, rather than because slow infusion caused headaches (39). Reductions in the infusion rate are a standard non-pharmacologic intervention to reduce the risk of headache and manage headache if it occurs during an infusion (13). Our results suggest that a shorter infusion time with a 15-minute rate escalation protocol is feasible for many patients. Patients’ sensitivity to the infusion rate may vary, and slower infusion rates may benefit patients with a history of frequent IVIG-related headache.

This pooled analysis is limited by the open-label design of the studies that were pooled for the analysis, the retrospective nature of the analysis, and the relatively small number of patients included, although the data set includes a large number of infusions. The pediatric patients included in trials GMX04 and GMX07 may not have reported their headache and migraine complaints appropriately. Lack of randomization increases the risk of bias in the subgroup analyses. In addition, the data are drawn from clinical trials rather than routine practice. Because of these limitations, the results should be considered suggestive rather than conclusive. However, the information presented here may help clinicians mitigate headache and migraine AEs in patients receiving IVIG infusions for PI.

In summary, this retrospective data analysis found in patients being treated with IVIG for PI, rates of headache (6.1%) and migraine (0.5%) were low for adult and pediatric patients and for both the 5% and 10% formulations. A large majority of infusions (94.6%) achieved the maximum infusion rate. Either formulation of this IVIG product can be administered using a 15-minute rate escalation protocol without excessive rates of headache or migraine.

## Data availability statement

The datasets presented in this article are not readily available because the datasets generated during and/or analyzed during the current study are not publicly available because they containing information that could compromise research participant privacy/consent. Reasonable requests as appropriate and permissible will be considered by the corresponding author. Requests to access the datasets should be directed to Kim Clark, Kim.Clark@bpl.co.uk.

## Ethics statement

The studies involving human participants were reviewed and approved by the institutional review board/ethics committee at each study center. The 3 trials each involved multiple centers. Participants from the included studies agreed to have their results published. Written informed consent to participate in this study was provided by each participant or the participants’ legal guardian/next of kin as applicable and appropriate.

## Author contributions

EW, KC, and BG contributed substantially to data collection, analysis, manuscript writing, and editing. ME conducted data analysis. All authors contributed to the manuscript and approved the submitted version.
