# Corrigendum: Low rates of headache and migraine associated with intravenous immunoglobulin infusion using a 15-minute rate escalation protocol in 123 patients with primary immunodeficiency

**DOI:** 10.3389/fimmu.2024.1430313

**Published:** 2024-05-29

**Authors:** Bob Geng, Kim Clark, Mark Evangelista, Eric Wolford

**Affiliations:** ^1^ Division of Allergy & Immunology, University of California, San Diego, CA, United States; ^2^ Global Medical Department, Bio Products Laboratory, Ltd., Elstree, United Kingdom; ^3^ Biostatistics Department, Atlantic Research Group, Charlottesville, VA, United States

**Keywords:** IVIg, headache, migraine, primary immunodefciencies, rate escalation, pooled

## Error in figure/table caption

In the published article, there was an error in the caption for **Figure 4** as published. The current caption to **Figure 4** mistakenly describes the figure as depicting weeks on the x-axis when it is actually reporting number of infusions. The figure caption was displayed as “Incidence of product-related headache and migraine by infusion number, number of events (A) 5% IVIG formulation (108 patients for weeks 1–5, then 75 patients for weeks 6–18) (B) 10% IVIG formulation (48 patients)”. The corrected caption appears below.

**Figure 4:**
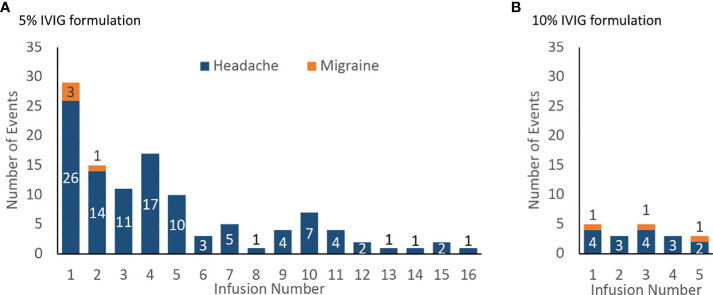
Incidence of product-related headache and migraine by infusion number, number of events **(A)** 5% IVIG formulation (108 patients for infusions 1–5, then 75 patients for infusions 6–18) **(B)** 10% IVIG formulation (48 patients).

Incidence of product-related headache and migraine by infusion number, number of events (A) 5% IVIG formulation (108 patients for infusions 1–5, then 75 patients for infusions 6–18) (B) 10% IVIG formulation (48 patients).

The authors apologize for this error and state that this does not change the scientific conclusions of the article in any way. The original article has been updated.

